# Longitudinal retinal microvascular changes and their association with white matter hyperintensities in neuromyelitis optica spectrum disorder

**DOI:** 10.3389/fneur.2026.1772477

**Published:** 2026-04-15

**Authors:** Yi Liu, Saichan Xu, Hao Lin, Xiaohong Lei, Yanping Li, Zhipeng Xu, Ruili Wei, Hui-Rong Jiang, Jianhui Mei

**Affiliations:** 1 Department of Neurology, QuZhou KeCheng People’s Hospital, Quzhou, Zhejiang, China; 2 Department of Critical Care Medicine, Yunhe People’s Hospital, Yunhe, Zhejiang, China; 3 The Fourth School of Clinical Medicine, Zhejiang Chinese Medical University, Hangzhou First People’s Hospital, Hangzhou, Zhejiang, China; 4 Department of Critical Care Medicine, The First Affiliated Hospital, Zhejiang University School of Medicine, Hangzhou, Zhejiang, China; 5 Department of Neurology, The First Affiliated Hospital, Zhejiang University School of Medicine, Hangzhou, Zhejiang, China; 6 Strathclyde Institute of Pharmacy and Biomedical Sciences, University of Strathclyde, Glasgow, United Kingdom

**Keywords:** neuromyelitis optica spectrum disorder, optical coherence tomography angiography, radial peripapillary capillary density, retinal microvasculature, white matter hyperintensity

## Abstract

**Background:**

Neuromyelitis optica spectrum disorder (NMOSD) is an autoimmune astrocytopathy characterized by recurrent inflammatory attacks within the central nervous system. Beyond optic neuritis, diffuse retinal and cerebral alterations may occur. Optical coherence tomography angiography (OCTA) enables the non-invasive assessment of retinal microvasculature and may provide insight into longitudinal retinal changes and their association with cerebral white matter hyperintensities (WMHs).

**Methods:**

In this longitudinal observational study, 45 aquaporin-4 antibody-positive NMOSD patients and 45 age- and sex-matched healthy controls underwent OCTA imaging. Radial peripapillary capillary (RPC) density was measured in the peripapillary region, while superficial vascular complex (SVC), deep vascular complex (DVC), and foveal avascular zone (FAZ) area were assessed in the macular region. Brain MRI was performed at baseline and at a 1-year follow-up, and WMH burden was rated using the Fazekas scale. Associations between OCTA metrics and WMHs were evaluated using regression models adjusted for age and disease duration, and longitudinal changes were analyzed using mixed-effects models.

**Results:**

At baseline, NMOSD eyes, including those without prior optic neuritis, showed significantly reduced RPC, SVC, and DVC densities and an enlarged FAZ area compared with controls (all *p* < 0.0001). Lower RPC density was associated with higher periventricular and total WMH burden (standardized *β* range: −0.45 to −0.52, all *p* < 0.01). Over 1 year, RPC and SVC densities declined further (both *p* < 0.0001), whereas DVC density and FAZ area remained stable. RPC density demonstrated the highest discriminatory performance in ROC analyses (AUC = 0.78).

**Conclusion:**

OCTA reveals dynamic retinal microvascular alterations in NMOSD and a consistent association between reduced peripapillary capillary density and WMH burden. These findings are observational and do not imply causality, but they support the use of OCTA as a sensitive tool for monitoring retinal involvement in NMOSD.

## Introduction

Neuromyelitis optica spectrum disorder (NMOSD) is a severe autoimmune astrocytopathy characterized by recurrent inflammatory attacks targeting the optic nerves, spinal cord, and selected brain regions (1–3). NMOSD is primarily mediated by pathogenic immunoglobulin G antibodies against aquaporin-4 (AQP4-IgG), leading to astrocytic injury, secondary demyelination, and neuroaxonal loss (4). Although NMOSD is classically regarded as an inflammatory demyelinating disorder, accumulating evidence indicates that vascular dysfunction and microcirculatory impairment are integral to its pathophysiology and may contribute to both focal lesion development and diffuse neurodegeneration (5).

White matter hyperintensities (WMHs), a radiological hallmark of cerebral small vessel disease and chronic hypoperfusion, are being increasingly recognized in NMOSD (6–8), even in patients without conventional vascular risk factors. The WMH burden in NMOSD has been associated with cognitive dysfunction, disability accumulation, and disease duration, suggesting that microvascular injury may represent a parallel and clinically relevant pathological process distinct from acute inflammatory relapses. However, the mechanisms underlying WMH development in NMOSD remain poorly understood, and *in vivo* biomarkers that capture cerebral microvascular dysfunction longitudinally are lacking.

The retina offers a unique and accessible window into the central nervous system microvasculature (9). Sharing a common embryological origin, anatomical organization, and regulatory mechanisms with the brain, the retinal microvasculature mirrors cerebral small vessel pathology (10, 11). Optical coherence tomography angiography (OCTA) enables non-invasive, layer-specific visualization and quantification of retinal capillary plexuses, providing a powerful tool for assessing neurovascular integrity *in vivo* (12). In NMOSD, OCTA studies have consistently demonstrated reduced vessel density in the radial peripapillary capillary (RPC) network and macular vascular plexuses, as well as enlargement of the foveal avascular zone (1, 2, 13). Importantly, these alterations have been observed not only in eyes with a history of optic neuritis (ON) but also in clinically unaffected eyes, indicating the presence of subclinical and potentially systemic microvascular involvement (14, 15).

Among retinal vascular layers, the RPC network—supplying the retinal nerve fiber layer and closely coupled to astrocytic and Müller cell function—appears particularly vulnerable in NMOSD (16). Given the central role of AQP4-expressing glial cells in maintaining neurovascular homeostasis, RPC impairment may reflect AQP4-IgG-mediated disruption of the neurovascular unit, leading to chronic hypoperfusion and axonal degeneration. While cross-sectional studies in non-NMOSD populations have linked retinal microvascular changes to WMH burden (17, 18), whether a similar retinal-cerebral vascular coupling exists in NMOSD and whether such associations persist over time remain unknown.

In this prospective observational longitudinal study, we investigated retinal microvascular changes in AQP4-IgG-positive NMOSD using OCTA and examined their relationship with cerebral WMH burden assessed by magnetic resonance imaging. We specifically aimed to (i) characterize longitudinal alterations in retinal capillary plexuses in NMOSD eyes with and without prior optic neuritis and (ii) determine whether retinal microvascular metrics are associated with the severity and progression of WMHs. By integrating retinal and cerebral imaging biomarkers, this study seeks to elucidate shared microvascular mechanisms underlying NMOSD-related neurodegeneration and to evaluate the potential of OCTA-derived measures as non-invasive markers for monitoring disease progression.

## Methods

### Study design

This single-center, observational study was conducted at the First Affiliated Hospital, Zhejiang University School of Medicine, China, from September 2021 to December 2024. The study protocol was approved by the Institutional Ethical Review Board of The First Affiliated Hospital of Zhejiang University (No. IIT2021-1163) and adhered to the Declaration of Helsinki. All participants provided written informed consent before enrollment.

### Participants

A total of 45 NMOSD patients were enrolled and followed at the Neurology Department of the First Affiliated Hospital, Zhejiang University School of Medicine. Inclusion criteria included a definitive diagnosis of AQP4-ab seropositive NMOSD according to the 2015 international consensus criteria (19) and complete longitudinal clinical and OCTA imaging with 1-year follow-up and an age between 18 and 50 years at baseline. Exclusion criteria included ocular disorders affecting OCTA data, such as severe glaucoma, age-related macular degeneration, poor OCTA image quality, and other confounding neurological disorders. Clinical data, including the Expanded Disability Status Scale (EDSS), disease duration, and ON frequency, were recorded.

Age- and sex-matched healthy controls were selected who had no history of ophthalmic, neurological, or psychological disorders and were aged 18 years or older.

All participants underwent comprehensive ocular examinations, including slit-lamp biomicroscopy and fundoscopy, to exclude preexisting ocular disorders. Intraocular pressure (IOP) was measured and compared between NMOSD patients and controls to confirm comparable baseline ocular health. Visual acuity was assessed using the Snellen chart. Continuous adherence to stable disease-modifying therapy (DMT) for ≥6 months before enrollment is required for all NMOSD patients.

### Spectral domain OCTA

OCTA imaging was performed using the Avanti RTVue-XR (Optovue, Fremont, California, United States; software version 2017.100.0.1), with an axial scan speed of 100 kHz, using an 840-nm wavelength laser with a tuning range of 100 nm. The image resolution was 5.3 mm axially and 18 mm laterally.

OCTA images were acquired using Avanti RTVue-XR, using a split-spectrum amplitude decorrelation algorithm. To evaluate peripapillary perfusion, cross-sectional registered reflectance intensity and flow images were summarized and viewed as an en face maximum flow projection. The radial peripapillary capillary (RPC) network was assessed within a 0.7-mm wide elliptical annular region extending outward from the optic disk boundary, with vasculature in the internal limiting membrane (ILM) and retinal nerve fiber layer (RNFL) analyzed automatically using in-built software. The software segmented the macula into the superficial vascular complex (SVC) and deep vascular complex (DVC), defined by the inner two-thirds and outer one-third of the ganglion cell layer and inner plexiform layer (IPL) (Supplementary Figure 1). A parafoveal capillary network was mapped within the annular zone diameter (0.6–2.5 mm) around the fovea. Vessel densities, representing the percentage (%) of large vessels and microvasculature in the analyzed region, were automatically generated across the entire scan area. The foveal avascular zone (FAZ) was automatically determined by the OCTA tool. The OCTA data collected in our study adhered to the OSCAR-MP quality criteria (20) and the APOSTEL recommendation (21). All images were reviewed by an ophthalmologist blinded to the subject’s diagnostic category.

### MR imaging

A standardized protocol was conducted using a 3.0 T MRI system (SIGNA MR, GE Healthcare, WI, United States) for all participants, including 3D T1-weighted images, axial T2-weighted images, and axial T2 FLAIR images. For the present study, the high-resolution T1-weighted images were acquired using a magnetization-prepared rapid gradient-echo (MPRAGE) sequence with the following parameters: echo time (TE) = 2.19 ms, repetition time (TR) = 2000 ms, inversion time = 900 ms, field of view (FOV) = 240 mm × 240 mm, matrix size = 256 × 256, flip angle = 8°, and voxel size = 0.9 mm × 0.9375 mm × 0.9375 mm.

WMHs were rated according to the Standards for Reporting Vascular Changes on Neuroimaging consensus criteria (22). WMHs were rated on FLAIR images using the Fazekas scale (23). WMH severity was rated (0–3) separately for periventricular (PWMH) and deep (DWMH) regions of the brain, with the sum of scores representing the total (TWMH) WMH burden (Supplementary Figure 2).

### Statistical analyses

Imaging data that passed quality assurance checks were used to generate derived metrics, and tabular data were screened for outliers, with values greater than three standard deviations (SDs) from the mean excluded from further analysis. Results are presented as mean (± SD) or number (%) in tables, unless otherwise noted. To assess group differences (controls vs. NMOSD) in demographic characteristics, the chi-squared test was applied to categorical variables, the two-sample *t*-test to continuous variables between two groups, and one-way analysis of variance (ANOVA) to continuous variables with three groups.

Baseline differences in OCTA metrics and visual acuity between groups were analyzed pairwise using generalized estimating equation (GEE) models to account for inter-eye within-patient correlations of monocular measurements. Longitudinal analysis of OCTA metrics was performed using linear mixed-effects models, incorporating time from baseline and group as fixed effects, and patient ID and eye (OD and OS) as random effects. Results are reported for the effect “time from baseline×group,” which reflects the OCTA metric change over time.

Logistic regression models were used to analyze the association between OCTA metrics and WMHs while adjusting for age and duration. Model estimates for the continuous predictors are presented as standardized betas [(*β*), per standard deviation (SD) increase/decrease].

The diagnostic potential of OCTA metrics in NMOSD patients relative to controls was assessed using the area under the receiver operating characteristic (AUROC) calculation. Furthermore, the diagnostic potential of OCTA metrics to detect microvascular changes (NMOSD at baseline and NMOSD at follow-up) in NMOSD patients was assessed. An AUROC of 1.0 denotes perfect discrimination, while a value of 0.5 denotes random discrimination.

Statistical significance was set at *p* < 0.05. We performed all analyses in R version 4.4.2 using the gee and dplyr packages, and generated plots with ggplot2.

## Results

**Figure 1:**
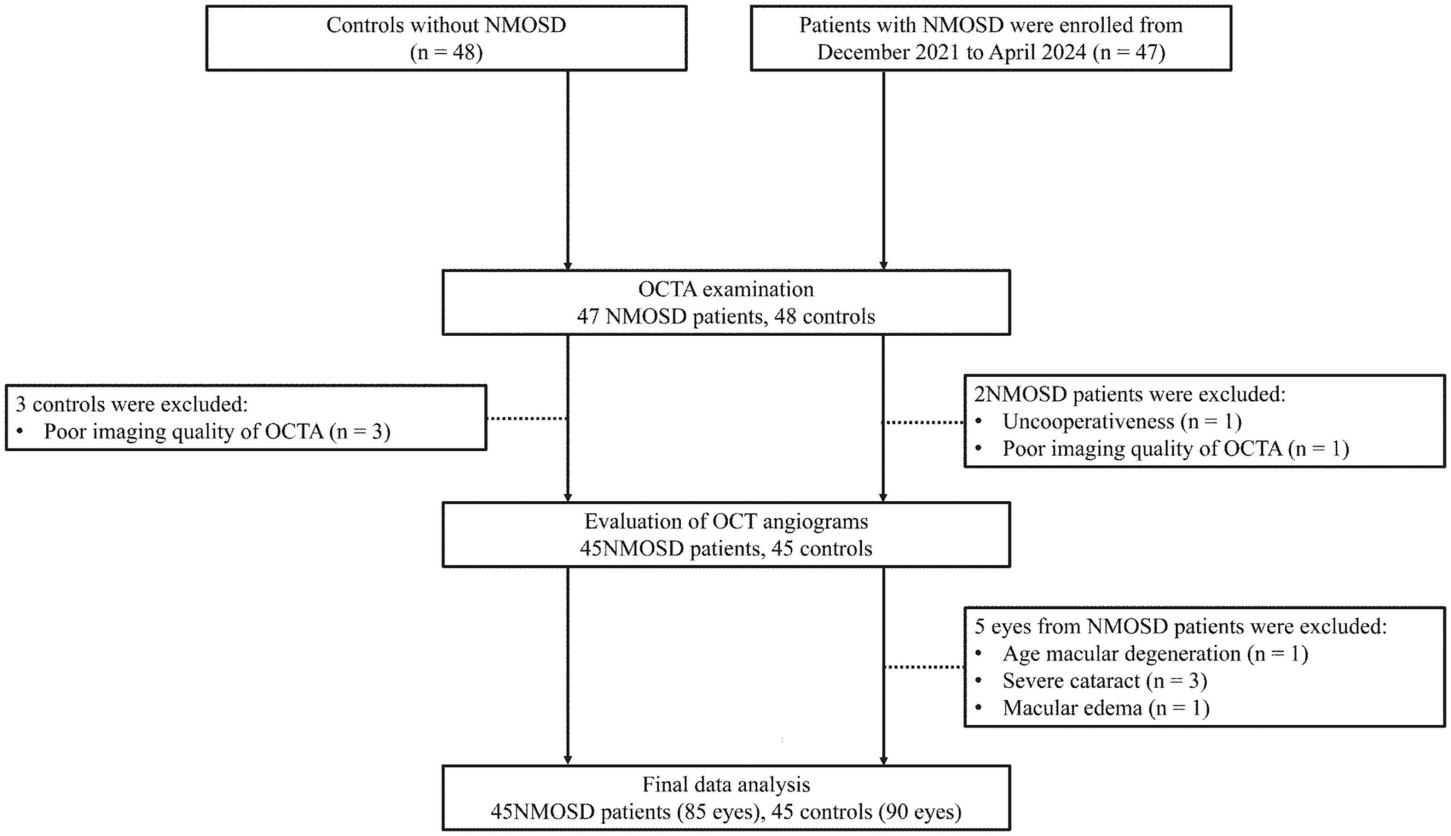
Flowchart of participant selection and exclusion.

A total of 47 NMOSD patients and 48 controls underwent OCTA examination; after excluding 3 controls (poor imaging quality) and 2 NMOSD patients (1 uncooperative, 1 poor OCTA quality), 45 NMOSD patients and 45 controls underwent OCT angiogram evaluation. Five eyes from NMOSD patients were further excluded due to confounding ocular conditions (1 age-related macular degeneration, 3 severe cataracts, and 1 macular edema), resulting in a final analytic cohort of 45 NMOSD patients (85 eyes) and 45 controls (90 eyes) (Figure 1).

At baseline, the NMOSD group comprised 45 individuals (mean age: 42.89 ± 11.28 years; 100% female), with 54 eyes affected by optic neuritis (NMOSD+ON) and 31 eyes without prior ON (NMOSD−ON); among them, 36 patients had unilateral ON and 9 had bilateral ON. Controls (mean age: 45.07 ± 9.79 years; 100% female) were age- and sex-matched. NMOSD patients exhibited significantly reduced best-corrected visual acuity compared with controls (all *p* < 0.001). At baseline, all 45 patients were maintained on stable DMT regimens (mean duration: 12.3 ± 5.7 months). The predominant DMT regimens were rituximab (62.2%), azathioprine/prednisone (15.6%), mycophenolate mofetil/prednisone (8.9%), inebrotumab (11.1% as a rituximab alternative due to infusion reactions), and satralizumab (2.2%). Notably, no patients received eculizumab or inebrotumab monotherapy without concomitant corticosteroid tapering. All 45 NMOSD patients maintained their initial DMT regimens throughout the 1-year follow-up period without any changes in therapy. No patients experienced treatment modifications or discontinuations during the study period. Baseline demographic and clinical characteristics are summarized in Table 1.

**Table 1:** Demographic and clinical information of study participants.

Characteristic	NMOSD (*n* = 45 patients, 85 eyes)	Controls (*n* = 45 subjects, 90 eyes)	*p*
Demographics			
Age, years, mean ± SD	42.89 ± 11.28	45.07 ± 9.79	0.268
Female, *n* (%)	45 (100.0%)	45 (100.0%)	–
Disease subgroup (Eyes)			
Total eyes, *n*	85	90	–
NMOSD with ON (NMOSD+ON), *n* (%)	54 (63.5%)	–	–
NMOSD without ON (NMOSD-ON), *n* (%)	31 (36.5%)	–	–
Clinical and treatment characteristics (Patients)			
Disease duration, years, mean ± SD	5.2 ± 4.1	–	–
EDSS score, mean ± SD	3.5 ± 2.0	–	–
Patients on DMT, *n* (%)	45 (100%)	–	–
DMT regimen, *n* (%)			
Rituximab (anti-CD20)	28 (62.2%)	–	–
Inebotuzumab (off-label anti-CD20)	5 (11.1%)	–	–
Azathioprine + Prednisone	7 (15.6%)	–	–
Mycophenolate mofetil + Prednisone	4 (8.9%)	–	–
Satralizumab (anti-IL-6R, SC)	1 (2.2%)	–	–
ON laterality (Patients)			
Patients with unilateral ON, *n* (%)	36 (80.0%)	–	–
Patients with bilateral ON, *n* (%)	9 (20.0%)	–	–
Visual function (Eyes)			
Visual acuity (LogMAR), mean ± SD	0.11 ± 0.25	−0.05 ± 0.07	<0.05*

LogMAR, Logarithm of Minimum Angle of Resolution; ON, Optic Neuritis; EDSS, Expanded Disability Status Scale; DMT, Disease-Modifying Therapy.

* Indicates a statistically significant difference.

### OCTA group differences at baseline

**Figure 2:**
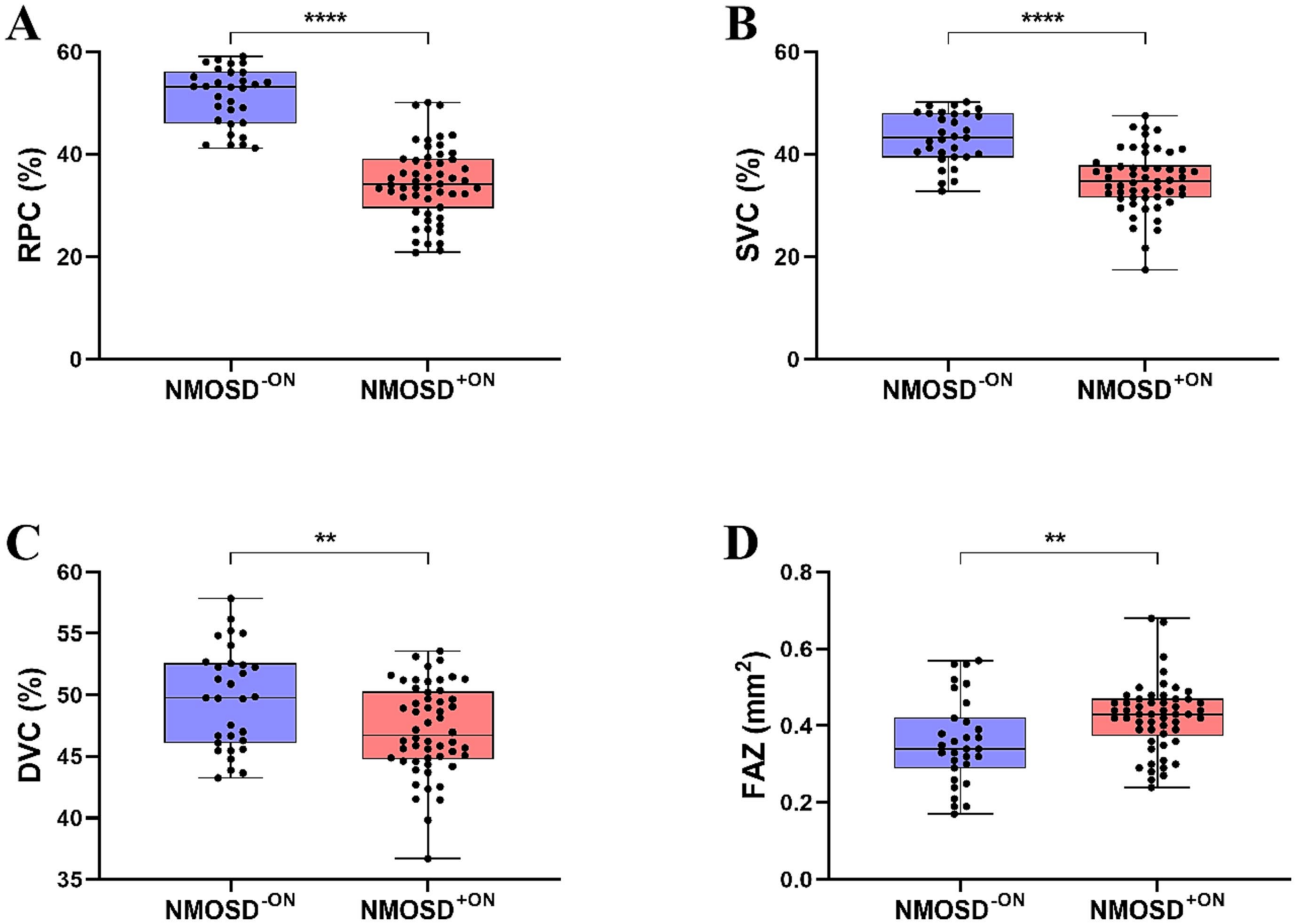
Comparison of OCTA metrics in NMOSD patients with and without ON at baseline.

First, we analyzed group differences at baseline between controls and NMOSD (mix of −ON and +ON). We observed that NMOSD patients had significantly reduced microvascular densities (RPC, SVC, and DVC) and significantly enlarged FAZ area compared to controls (all *p* < 0.0001). Similarly, NMOSD eyes with and without ON showed significantly reduced lower retinal microvasculature and enlarged FAZ compared to controls, respectively (*p* < 0.05). Supplementary Figure 2 shows the difference in OCTA metrics between controls and NMOSD at baseline. Our ROC analysis showed that RPC density had the highest AUC (AUC = 0.782, 95% CI 0.685–0.881) among all OCTA measures for detecting microvascular changes in NMOSD and controls. DVC showed the highest AUC (AUC = 0.755) among all OCTA measures for detecting microvascular changes in differentiating NMOSD eyes without ON and controls, as shown in Supplementary Table 4 (Figure 2).

**Figure 3:**
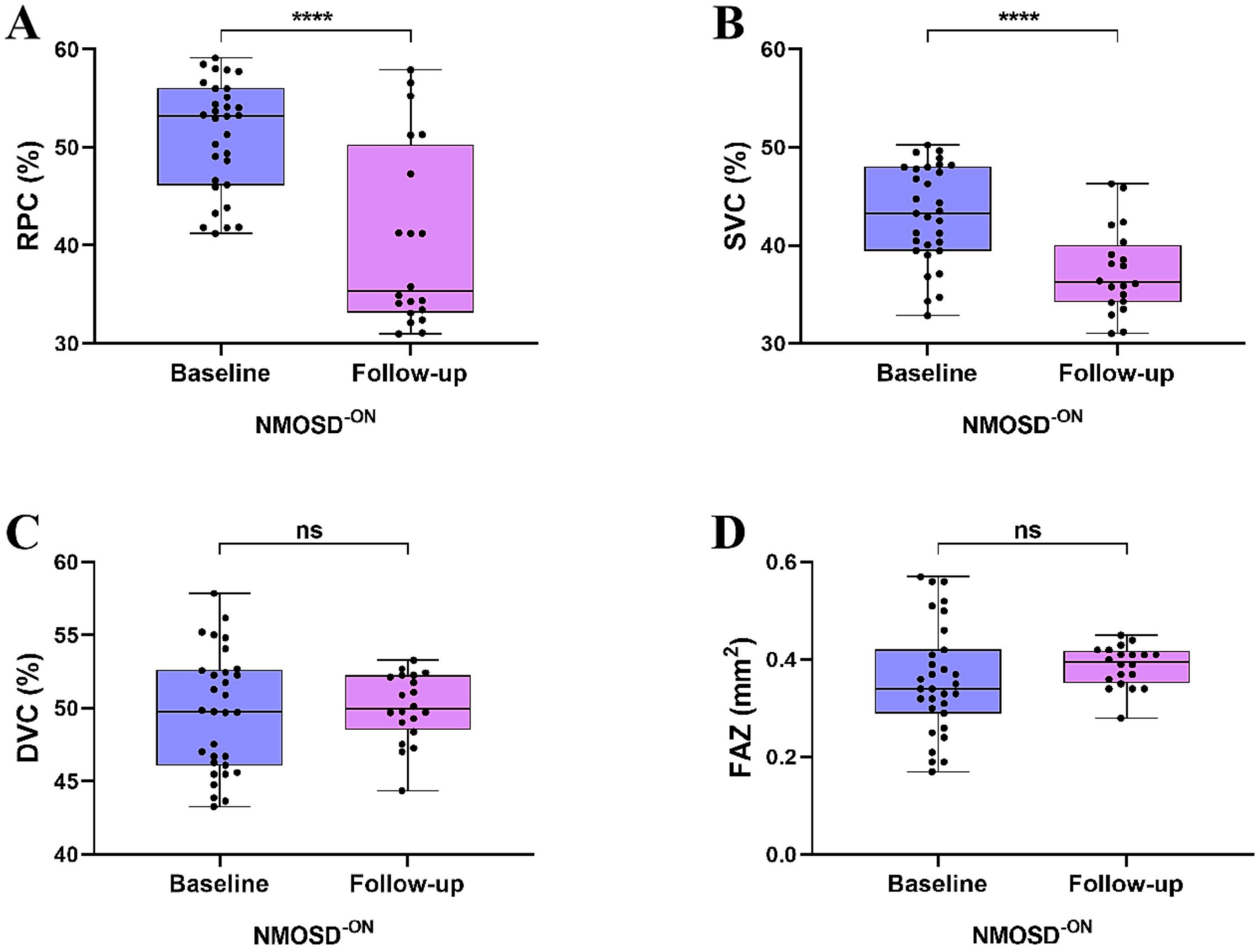
Comparison of OCTA metrics in NMOSD patients at baseline and 1-year follow-up.

Second, we analyzed the intergroup differences between NMOSD^-ON^ and NMOSD^+ON.^ NMOSD^+ON^ eyes had lower RPC (*p* < 0.001), SVC (*p* < 0.001), and DVC (*p* < 0.01) densities in comparison to NMOSD^-ON^ eyes; as expected, the FAZ area was larger in NMOSD^+ON^ eyes compared to NMOSD^-ON^ eyes (*p* < 0.01). Figure 3 shows a comparison of OCTA metrics of eyes between the NMOSD^-ON^ and NMOSD^+ON^ groups.

### Association between OCTA metrics and WMH burden in NMOSD at baseline

At baseline, RPC was associated with PWMH in NMOSD^-ON^ (*β* = −0.523, *p* = 0.003, Supplementary Figure 4). No significant association was seen in the SVC and DVC with WMH burden in NMOSD^-ON^. In NMOSD^+ON^, reduced RPC density and enlarged FAZ area were associated with increased PWMH and TWMH burden (all *p* < 0.001). No significant association was seen in the SVC and DVC with WMH burden in NMOSD^+ON^.

### OCTA changes during follow-up

Two NMOSD patients were excluded from follow-up due to blindness, leaving 43 NMOSD patients who were included and analyzed in our follow-up study. Within a year, 11 NMOSD patients who initially presented with unilateral ON subsequently progressed to bilateral ON involvement. Our follow-up study included 83 eyes from 43 NMOSD patients, with 63 eyes having ON (NMOSD^+ON^) and 20 eyes without ON (NMOSD^-ON^).

**Figure 4:**
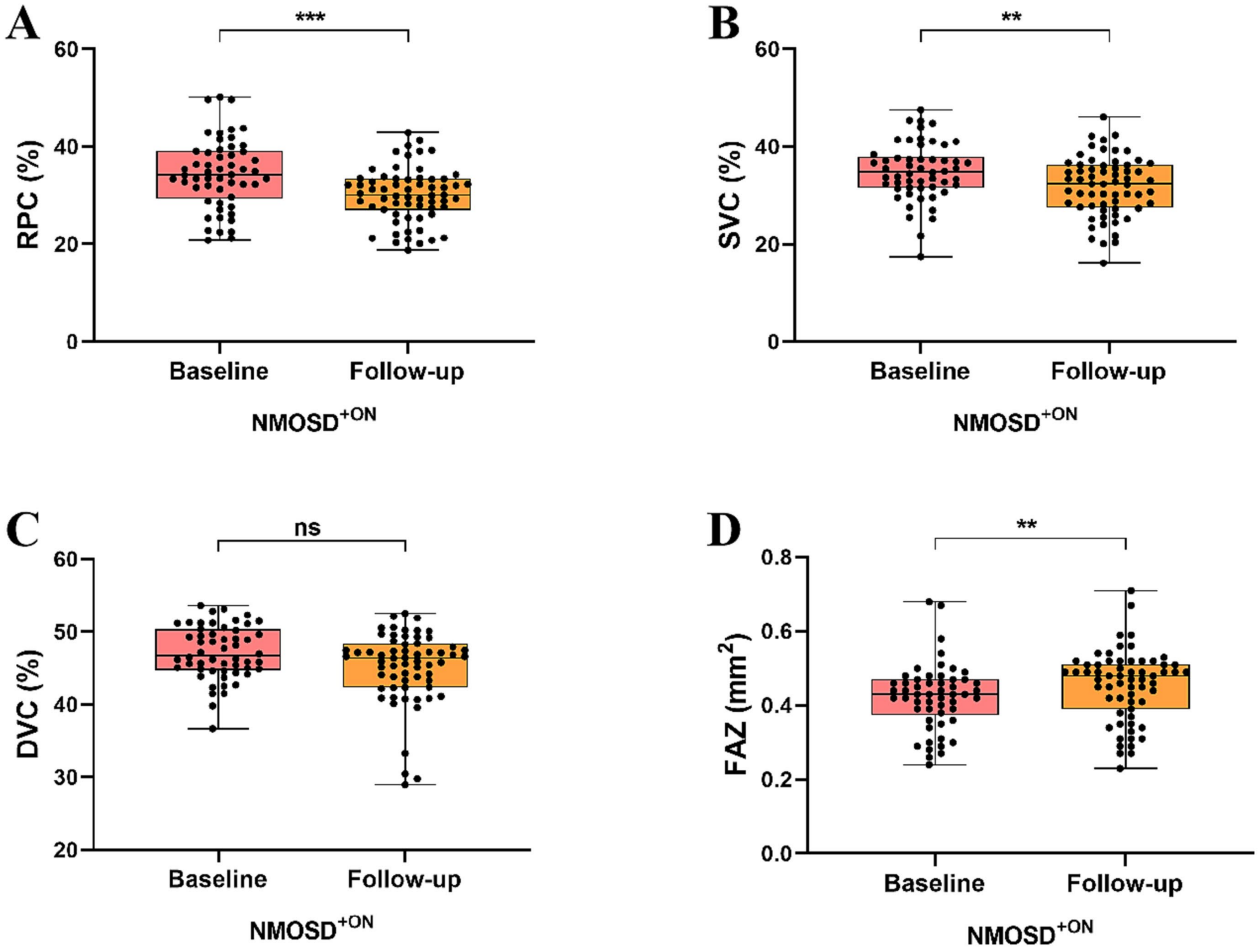
Comparison of OCTA metrics in NMOSD patients with ON at baseline and follow-up.

**Figure 5:**
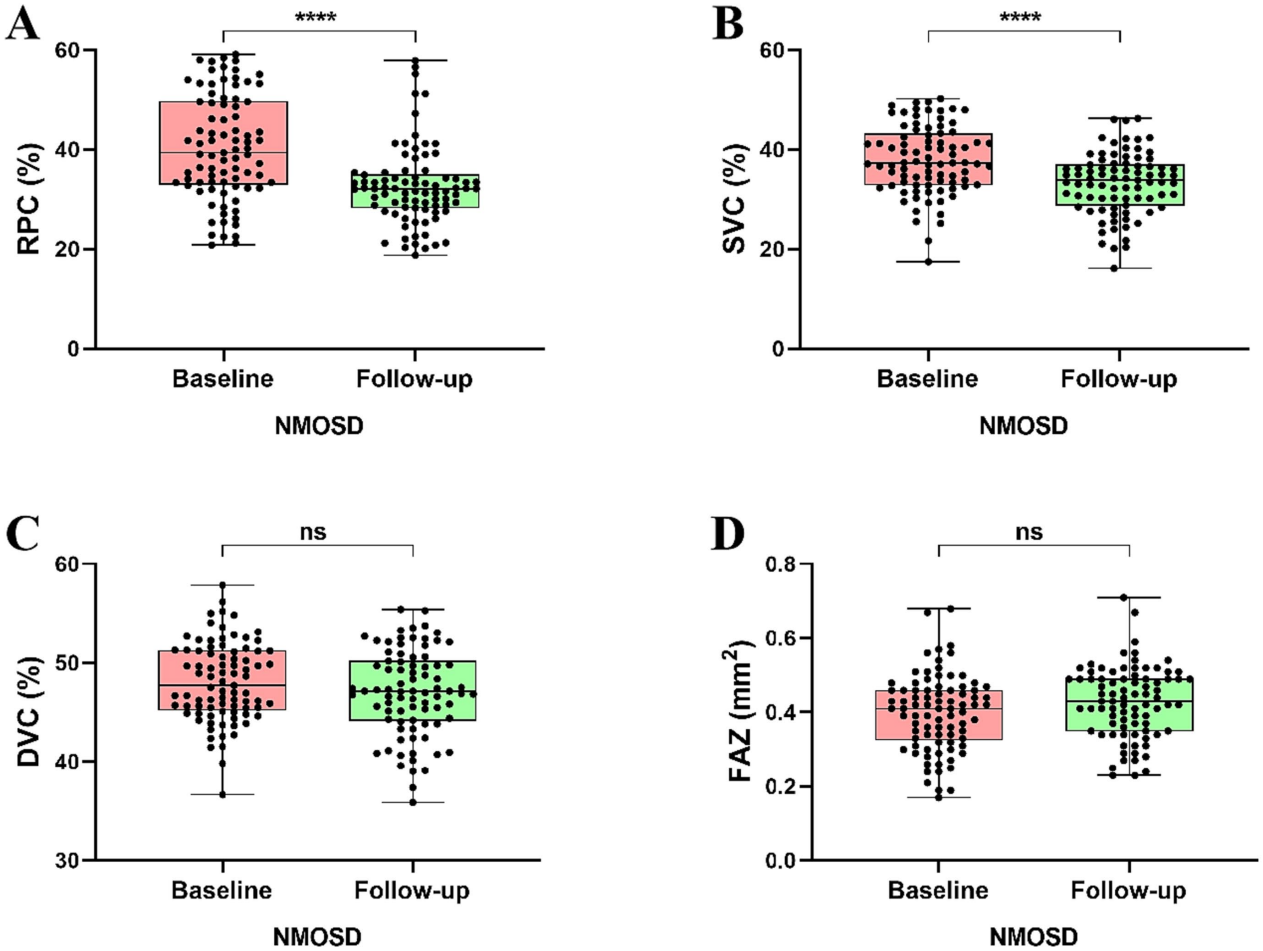
Comparison of OCTA metrics in NMOSD patients without ON at baseline and follow-up.

Longitudinal analysis showed a significant reduction in the RPC (*p* < 0.0001) and SVC (*p* < 0.0001) densities in all NMOSD patients compared to baseline analysis. However, no significant differences were observed in the DVC density (*p* = 0.228) and FAZ area (*p* = 0.051), as shown in Figure 3. On follow-up, we found that RPC (*p* < 0.001) and SVC (*p* < 0.01) densities were significantly reduced, and FAZ area (*p* < 0.01) was significantly enlarged in NMOSD^+ON^ patients than in NMOSD^+ON^ at baseline; no significant difference was seen in the DVC density (*p* = 0.044), as shown in Figure 4. Similarly, we found that RPC and SVC (*p* < 0.0001) densities were significantly reduced in NMOSD^-ON^ patients on follow-up compared to NMOSD^-ON^ at baseline; no significant differences were seen in the DVC density (*p* = 0.749) and FAZ area (*p* = 0.090), as shown in Figure 5.

### Association between OCTA metrics and WMH burden in NMOSD at follow-up

We found that reduced RPC density was associated with increased PWMH and TWMH in NMOSD^-ON^ and NMOSD^+ON,^ respectively (all *p* < 0.001, Supplementary Figure 5). No significant association was seen in the SVC and DVC with WMH burden in NMOSD^-ON^ and NMOSD^+ON^, respectively (all *p* > 0.05).

### ROC analysis of OCTA metrics

**Figure 6:**
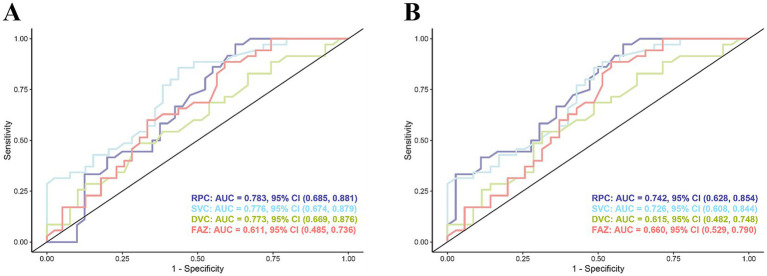
ROC analysis of OCTA metrics. **(A)** ROC analysis of OCTA metrics between NMOSD and controls. **(B)** OCTA metrics between baseline and follow-up. RPC shows the highest discriminatory AUC.

Using the OCTA metrics, RPC demonstrated the highest area under the curve [AUC = 0.783, 95% confidence interval (CI), 0.685–0.881, *p* < 0.001; Supplementary Table 1] among all metrics for detecting microvascular changes in NMOSD and controls. Similarly, RPC demonstrated the highest area under the curve [AUC = 0.992, 95% confidence interval (CI), 0.973–1.00, *p* < 0.001; Supplementary Table 1], among all metrics for detecting microvascular changes in NMOSD and controls (Figure 6A, Supplementary Table 1).

By comparing our follow-up and baseline OCTA analyses, RPC demonstrated the highest area under the curve (AUC = 0.742, 95% confidence interval [CI], 0.628–0.855, *p* < 0.001; Figure 6B) for detecting longitudinal microvascular damage in NMOSD.

## Discussion

In this longitudinal observational study, we investigated retinal microvascular alterations in AQP4-IgG-positive NMOSD using OCTA and examined their association with WMH burden. The principal findings are that (i) retinal microvascular density, particularly within the RPC network, was reduced in NMOSD compared with healthy controls, including in eyes without a history of ON; (ii) RPC and SVC densities showed further decline over 1 year, whereas DVC density and FAZ area remained relatively stable; and (iii) reduced RPC density was consistently associated with higher WMH burden at both baseline and follow-up. These findings demonstrate reproducible longitudinal associations between retinal microvascular metrics and cerebral WMHs in NMOSD, but they do not establish causality or directionality.

Our findings are consistent with prior cross-sectional OCTA studies reporting reduced retinal vessel density in NMOSD, both in eyes with previous ON and in clinically unaffected eyes (2, 13, 24, 25). The presence of retinal microvascular alterations in NMOSD–ON eyes supports the notion that retinal involvement in NMOSD is not solely a secondary consequence of clinically overt optic nerve inflammation (26). Rather, these changes may reflect diffuse or subclinical processes affecting the visual pathway or retinal neurovascular unit. Importantly, our longitudinal data extend previous work by demonstrating that reductions in RPC and SVC density can be detected over time, even in the absence of new ON episodes (3).

Among the evaluated vascular layers, the RPC network showed the most pronounced and consistent alterations. This finding aligns with earlier reports suggesting that the peripapillary microvasculature is particularly sensitive to pathological changes in NMOSD (2, 16). However, reduced vessel density on OCTA should not be interpreted as direct evidence of primary vascular injury. An alternative and equally plausible explanation is that reductions in capillary density reflect decreased metabolic demand secondary to retinal ganglion cell or axonal loss, which is well documented in NMOSD (27). Given the tight coupling between neuronal integrity and microvascular architecture in the retina, OCTA-derived vessel density changes likely represent a composite marker integrating vascular, glial, and neuroaxonal alterations.

We observed a consistent association between lower RPC density and higher WMH burden at both baseline and follow-up. WMHs are non-specific MRI markers reflecting heterogeneous pathological substrates, including gliosis, demyelination, axonal loss, and microstructural tissue rarefaction (28). Although WMH are classically linked to cerebral small vessel disease in aging populations (22), their presence in NMOSD should be interpreted cautiously and not equated with primary vascular pathology. The observed association between retinal microvascular measures and WMH burden does not imply a shared causal mechanism. Rather, it suggests that retinal OCTA metrics and cerebral WMHs may capture parallel manifestations of global or diffuse central nervous system injury in NMOSD. These associations may arise from common downstream effects of chronic inflammation, astrocytic dysfunction, or cumulative tissue damage, rather than from direct retinal–cerebral vascular coupling (4, 6, 29). Importantly, our study design does not allow inference regarding temporal ordering or mechanistic linkage between retinal changes and WMH development.

NMOSD is not considered a progressive neurodegenerative disease in the same sense as multiple sclerosis. Therefore, longitudinal changes observed in OCTA metrics should not be interpreted as evidence of relapse-independent disease progression (3, 30). Instead, the observed changes likely reflect the accumulation of tissue alterations over time, influenced by prior inflammatory attacks, subclinical injury, and individual variability in disease course. The absence of robust WMH progression over the 1-year follow-up further underscores the limited ability to model change–change relationships between retinal and cerebral imaging markers within the current timeframe.

RPC density demonstrated the highest discriminatory performance in differentiating NMOSD eyes from healthy controls and in distinguishing baseline from follow-up measurements. These results suggest that RPC may be a sensitive imaging marker for retinal alterations in NMOSD. However, the term “diagnostic” should be interpreted cautiously. OCTA metrics are not disease-specific and should not be viewed as standalone diagnostic tools. Rather, they may provide complementary information within a multimodal imaging framework, particularly for monitoring retinal involvement over time.

Immunosuppressive therapies and high-dose corticosteroids are integral to NMOSD management and may influence retinal or cerebral microvasculature (31). Due to heterogeneous treatment exposure and the limited sample size, we were unable to systematically assess treatment-related effects on OCTA metrics or WMH burden. Similarly, cardiovascular and metabolic risk factors were not formally controlled for and may act as residual confounders. These factors should be carefully addressed in future studies through stratified analyses or large, prospectively designed cohorts. This study has several limitations that warrant consideration. First, its observational design precludes causal inference regarding the relationship between retinal microvascular alterations and white matter hyperintensity (WMH) burden; the associations observed should therefore be interpreted as correlative rather than mechanistic. Second, WMH severity was assessed using the visual Fazekas rating scale, which, although widely used and clinically validated, may lack sensitivity to subtle or disease-specific microstructural changes that could be detected using quantitative volumetric or diffusion-based MRI metrics. Third, the 1-year follow-up may be insufficient to capture meaningful longitudinal progression of cerebral WMHs or to robustly model dynamic change—change relationships between retinal and cerebral imaging biomarkers. Fourth, treatment exposure was heterogeneous across participants, and modifications in disease-modifying therapy during follow-up were not controlled analytically, introducing potential confounding effects on both retinal vascular metrics and cerebral imaging outcomes. Fifth, OCTA-derived vessel density measurements are susceptible to technical influences, including signal strength variability, segmentation accuracy, and residual motion or projection artifacts, despite adherence to established quality control standards. Finally, the absence of longitudinal OCTA data in healthy controls limits the ability to disentangle disease-related microvascular changes from age-related physiological variation.

In summary, this longitudinal observational OCTA study demonstrates dynamic retinal microvascular alterations in NMOSD and identifies a consistent association between reduced RPC density and cerebral WMH burden. These findings support the utility of OCTA as a non-invasive tool for capturing retinal changes in NMOSD, while underscoring the need for cautious interpretation. Future studies combining long follow-up, quantitative MRI metrics, and comprehensive clinical and vascular risk profiling are required to clarify the biological significance of these associations and their potential role in disease monitoring.

## Data Availability

The original contributions presented in the study are included in the article/Supplementary material, further inquiries can be directed to the corresponding author/s.

## Supplementary material

Supplementary Figure 1 Segmentation of retinal microvascular plexuses. Radial peripapillary capillary (RPC) was defined as the vasculature within the internal limiting membrane (ILM) and the nerve fiber layer (NFL). The superficial vascular complex (SVC) and deep vascular complex (DVC) and defined as the interface of the inner two thirds and outer one third of the ganglion cell layer and IPL.

Supplementary Figure 2 Visual rating of white matter hyperintensities (WMH) using the Fazekas scale. (**A–D**) Subcortical deep white matter hyperintensity—0 score: no lesion (**A**); 1 score: punctate lesion (**B**); 2 scores: lesion fusion (**C**); 3 scores: large area fusion of lesion (**D**); (**E–H**) paraventricular WMH—0 score: no lesion (E); 1 score: pencil or false thin layer lesion (F); 2 scores: smooth halo (**G**). 3scores: irregular paraventricular high signal intensity extending to the deep white matter (**H**); the lesion scores of the two sites were added (minimum 0 scores, maximum 6 scores).

Supplementary Figure 3 Comparison of OCTA metrics between NMOSD and controls at baseline.

Supplementary Figure 4 Association between OCTA metrics and WMH burden in NMOSD at baseline.

Supplementary Figure 5 Association between OCTA metrics and WMH burden in NMOSD at follow-up.
