# Supplementary material for: Longitudinal retinal microvascular changes and their association with white matter hyperintensities in neuromyelitis optica spectrum disorder
Source: Front Neurol. 2026 Apr 15;17:1772477. doi: 10.3389/fneur.2026.1772477 (PMC13124516; doi:10.3389/fneur.2026.1772477)
Supplement: Supplementary file 1 [file Table_1.docx]

Supplementary Table 1. ROC analysis of NMOSD in eyes with and without ON

| Measure | AUC | Youden’s Index | Sensitivity | Specificity | Cutoff | PPV | NPV |
| --- | --- | --- | --- | --- | --- | --- | --- |
| RPC | 0.992 | 0.933 | 93.33 | 1 | 0.422 | 1 | 0.96 |
| SVC | 0.854 | 0.625 | 1 | 62.5 | -1.352 | 0.625 | 1 |
| DVC | 0.535 | 0.208 | 1 | 20.8 | -0.579 | 0.441 | 1 |
| FAZ | 0.524 | 0.167 | 66.67 | 50.0 | -0.453 | 0.294 | 0.545 |

Supplementary Table 2. ROC analysis of NMOSD in eyes without ON and Controls

| Measure | AUC | Youden’s Index | Sensitivity | Specificity | Cutoff | PPV | NPV |
| --- | --- | --- | --- | --- | --- | --- | --- |
| RPC | 0.633 | 0.25 | 91.7 | 0.333 | -0.931 | 0.423 | 0.882 |
| SVC | 0.664 | 0.378 | 66.7 | 71.1 | -0.734 | 0.552 | 0.80 |
| DVC | 0.755 | 0.472 | 58.3 | 88.89 | -0.182 | 0.737 | 0.80 |
| FAZ | 0.594 | 0.375 | 0.375 | 1 | -0.208 | 1 | 0.75 |
