# Supplementary figures and images for: Longitudinal retinal microvascular changes and their association with white matter hyperintensities in neuromyelitis optica spectrum disorder
Source: Front Neurol. 2026 Apr 15;17:1772477. doi: 10.3389/fneur.2026.1772477 (PMC13124516; doi:10.3389/fneur.2026.1772477)

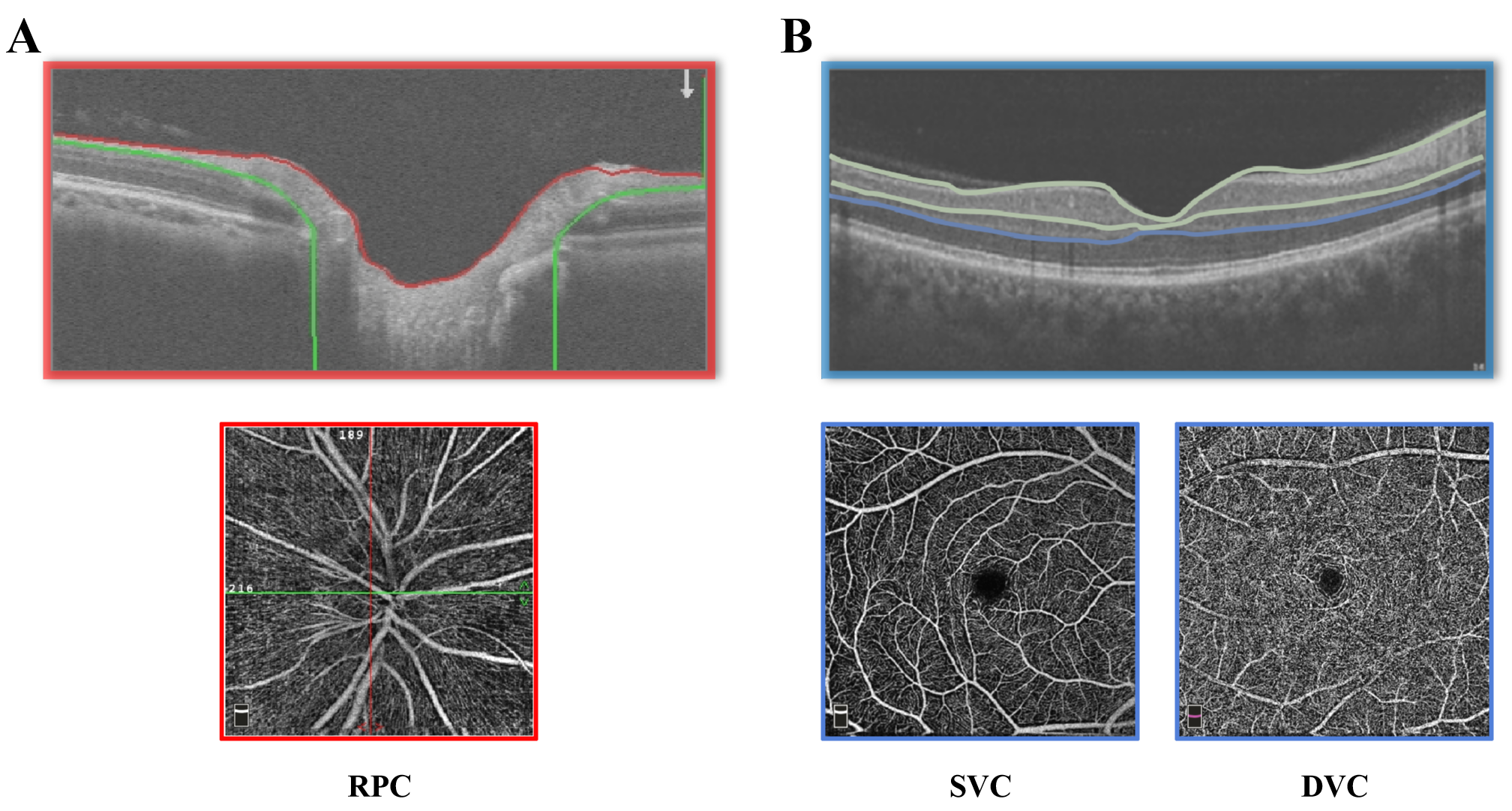

Supplement: Supplementary Figure 1 — Segmentation of retinal microvascular plexuses. Radial peripapillary capillary (RPC) was defined as the vasculature within the internal limiting membrane (ILM) and the nerve fiber layer (NFL). The superficial vascular complex (SVC) and deep vascular complex (DVC) and defined as the interface of the inner two thirds and outer one third of the ganglion cell layer and IPL. [file Image_1.tif]

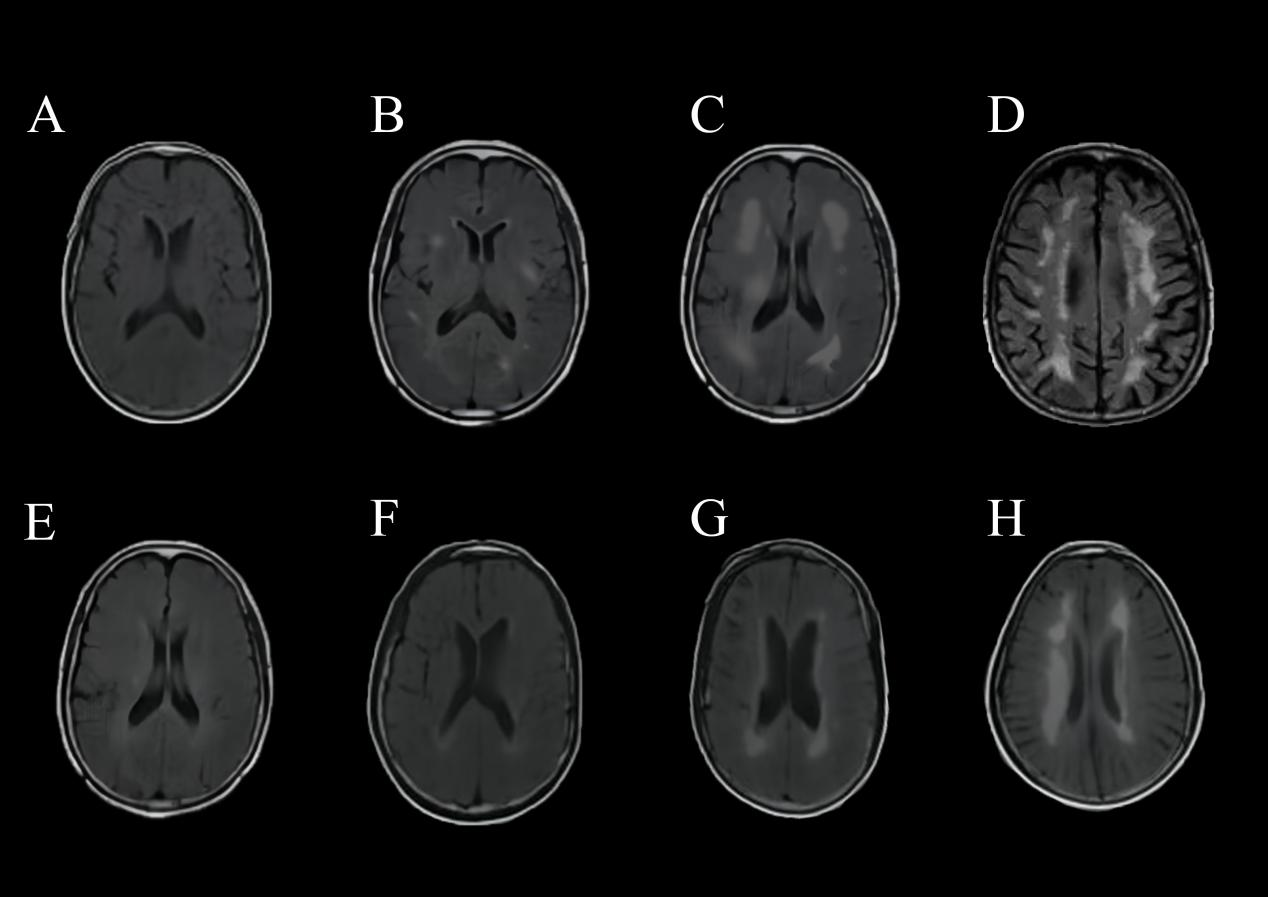

Supplement: Supplementary Figure 2 — Visual rating of white matter hyperintensities (WMH) using the Fazekas scale. (A–D) Subcortical deep white matter hyperintensity—0 score: no lesion (A); 1 score: punctate lesion (B); 2 scores: lesion fusion (C); 3 scores: large area fusion of lesion (D); (E–H) paraventricular WMH—0 score: no lesion (E); 1 score: pencil or false thin layer lesion (F); 2 scores: smooth halo (G). 3scores: irregular paraventricular high signal intensity extending to the deep white matter (H); the lesion scores of the two sites were added (minimum 0 scores, maximum 6 scores). [file Image_2.tif]

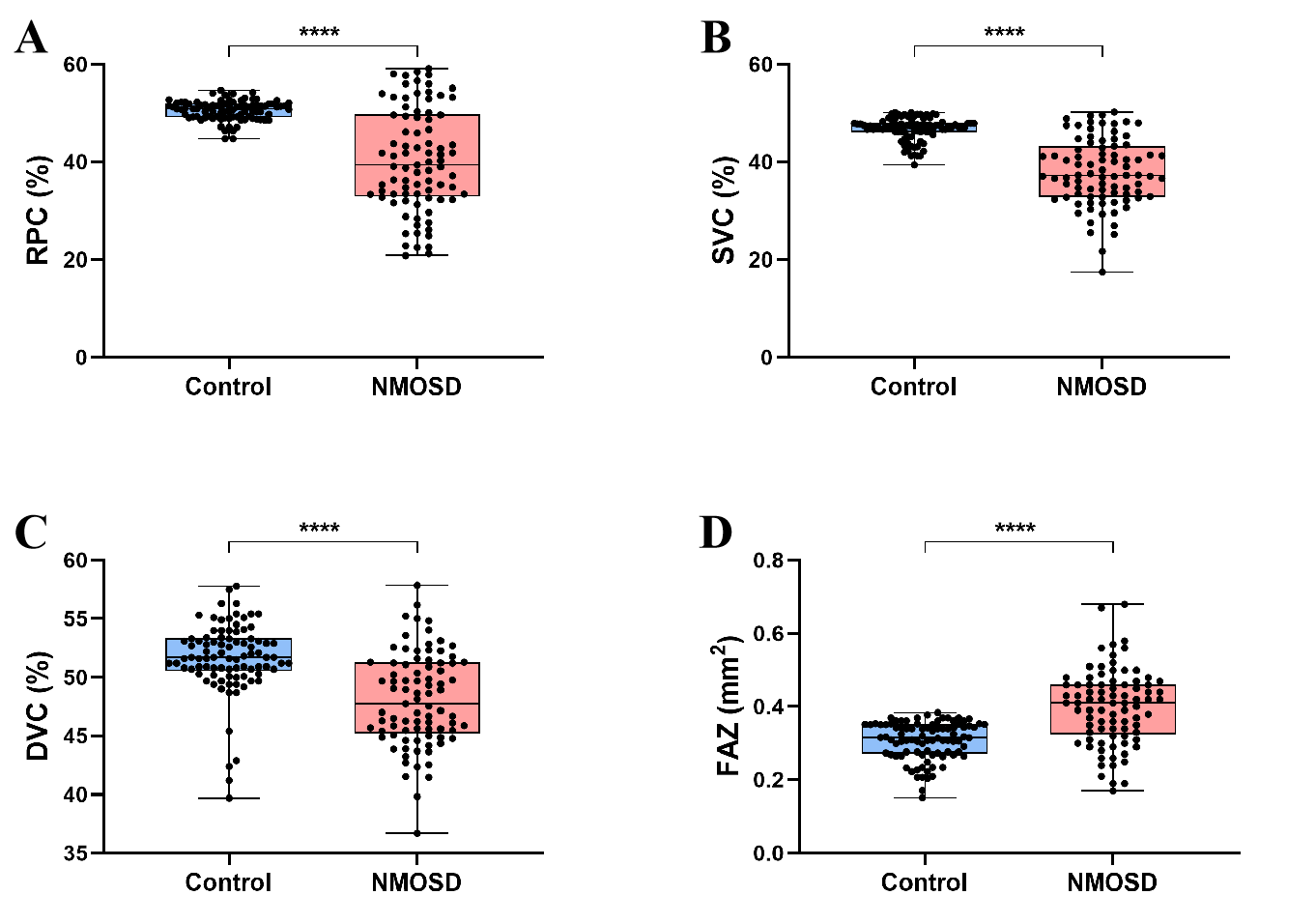

Supplement: Supplementary Figure 3 — Comparison of OCTA metrics between NMOSD and controls at baseline. [file Image_3.tif]

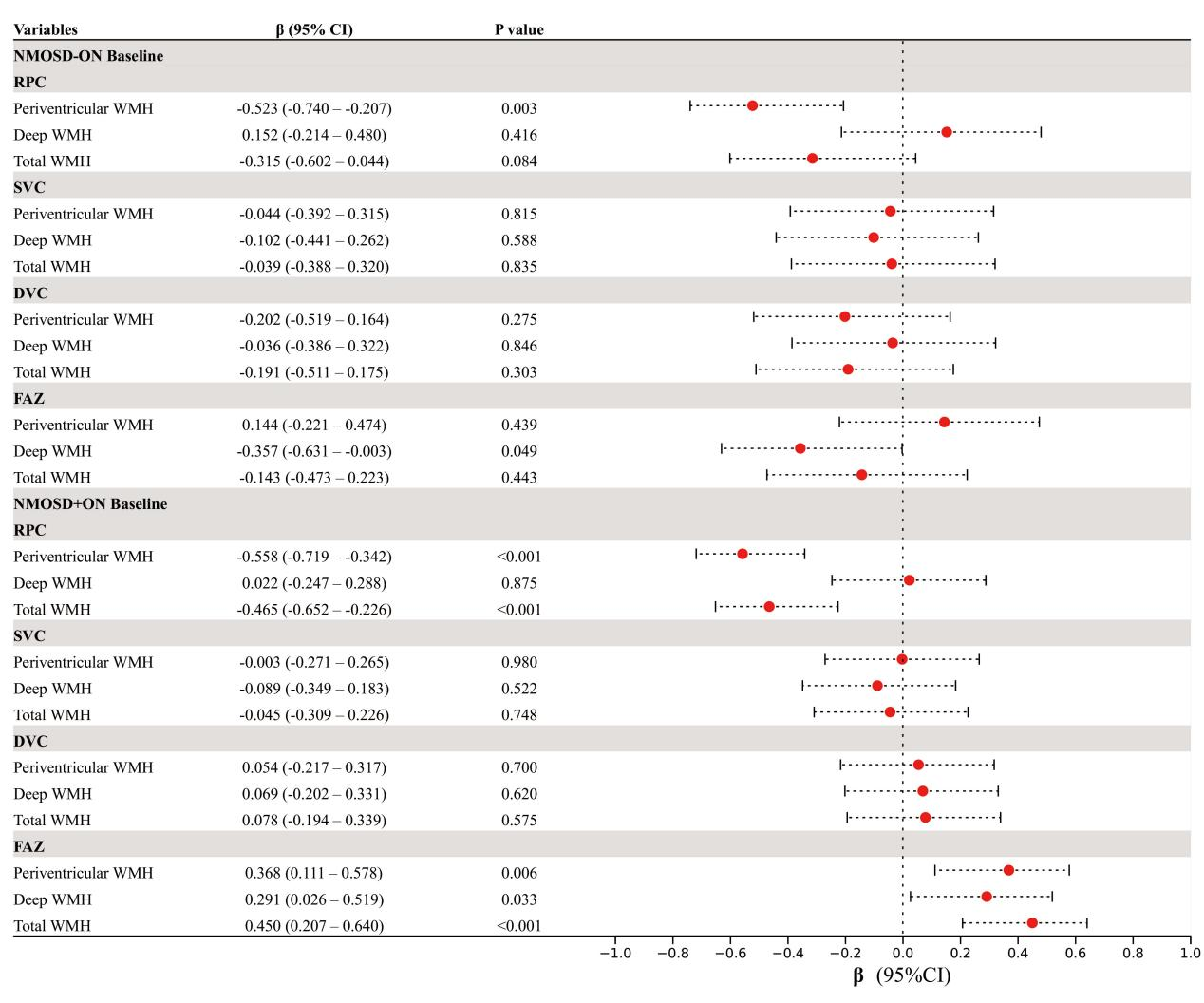

Supplement: Supplementary Figure 4 — Association between OCTA metrics and WMH burden in NMOSD at baseline. [file Image_4.tif]

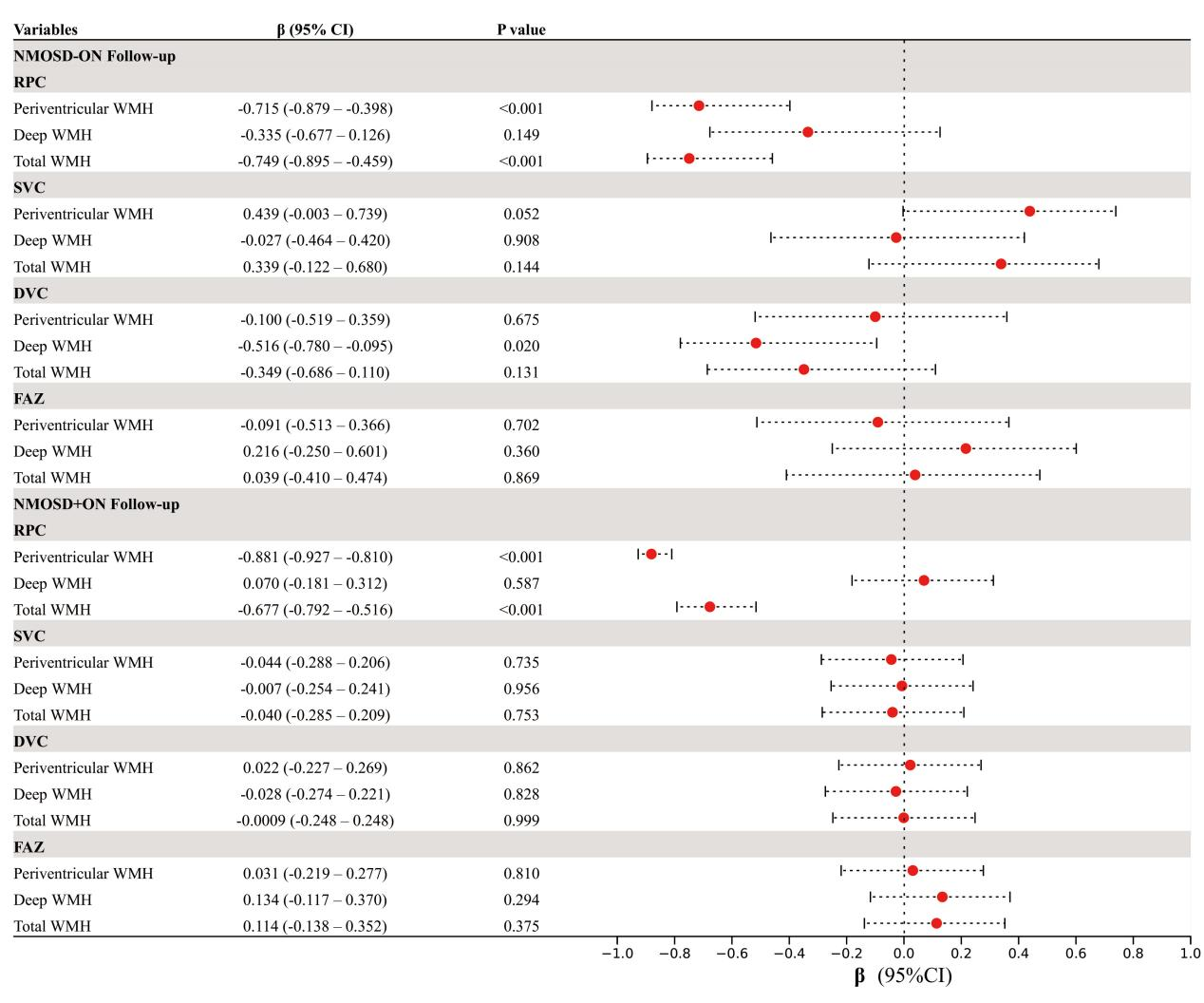

Supplement: Supplementary Figure 5 — Association between OCTA metrics and WMH burden in NMOSD at follow-up. [file Image_5.tif]
